# Follicular fluid placental growth factor is increased in polycystic ovarian syndrome: correlation with ovarian stimulation

**DOI:** 10.1186/1477-7827-12-82

**Published:** 2014-08-20

**Authors:** Reshef Tal, David B Seifer, Richard V Grazi, Henry E Malter

**Affiliations:** Division of Reproductive Endocrinology and Infertility, Genesis Fertility & Reproductive Medicine, Maimonides Medical Center, Brooklyn, NY USA

**Keywords:** Placental growth factor (PlGF), Soluble Fms-like tyrosine kinase (sFlt-1), Polycytic ovarian syndrome (PCOS), Angiogenesis, Ovarian stimulation

## Abstract

**Background:**

Polycystic ovarian syndrome (PCOS) is characterized by increased ovarian angiogenesis and vascularity. Accumulating evidence indicates that vascular endothelial growth factor (VEGF) is increased in PCOS and may play an important role in these vascular changes and the pathogenesis of this disease. Placental growth factor (PlGF), a VEGF family member, has not been previously characterized in PCOS women. We investigated levels and temporal expression patterns of PlGF and its soluble receptor sFlt-1 (soluble Fms-like tyrosine kinase) in serum and follicular fluid (FF) of women with PCOS during controlled ovarian stimulation.

**Methods:**

This was a prospective cohort study of 14 PCOS women (Rotterdam criteria) and 14 matched controls undergoing controlled ovarian stimulation. Serum was collected on day 3, day of hCG and day of oocyte retrieval. FF was collected on retrieval day. PlGF, sFlt-1 and anti-mullerian hormone (AMH) protein concentrations were measured using ELISA. Since sFlt-1 binds free PlGF, preventing its signal transduction, we calculated PlGF bioavailability as PlGF/sFlt-1 ratio.

**Results:**

Serum PlGF and sFlt-1 levels were constant throughout controlled ovarian stimulation, and no significant differences were observed in either factor in PCOS women compared with non-PCOS controls at all three measured time points. However, FF PlGF levels were increased 1.5-fold in PCOS women compared with controls (p < 0.01). Moreover, FF PlGF correlated positively with number of oocytes retrieved and the ovarian reserve marker anti-mullerian hormone (AMH) and negatively with age. In addition, FF sFlt-1 levels were decreased 1.4-fold in PCOS women compared to controls (p = 0.04). PlGF bioavailability in FF was significantly greater (2-fold) in PCOS women compared with non-PCOS controls (p < 0.01).

**Conclusions:**

These data provide evidence that FF PlGF correlates with ovarian stimulation and that its bioavailability is increased in women with PCOS undergoing controlled ovarian stimulation. This suggests that PlGF may play a role in PCOS pathogenesis and its angiogenic dysregulation.

## Background

Polycystic ovarian syndrome (PCOS) is a common endocrine disorder that affects 5-7% of women of reproductive age [1]. Its cardinal features are hyperandrogenism, oligoanovulation and polycystic ovaries [1]. PCOS presents itself with a wide spectrum of manifestations which may also include obesity, hyperlipidemia, insulin resistance, type II diabetes, and possibly cardiovascular disease [2, 3].

The pathogenesis of PCOS is not well understood but accumulating evidence suggests that dysregulation of angiogenic factors may play an important role. Vascular endothelial growth factor (VEGF) is the prototypical member of a family of angiogenic factors which includes angiopoietins, placental growth factor (PlGF), basic fibroblast growth factor (bFGF) and transforming growth factor-β1 (TGF-β1). While we and others have shown angiopoietins [4], bFGF [5] and TGF-β1 [6, 7] to be upregulated in PCOS, VEGF is the most extensively studied angiogenic factor in connection with PCOS pathophysiology [8]. VEGF is one of the major regulators of angiogenesis and its important role has been demonstrated in developmental, physiological and pathological angiogenesis [9]. It is a heparin-binding homodimeric protein of 46 kDa that consists of six isoforms, and is a potent mitogen for endothelial cells [9, 10]. Its action is mediated by binding to tyrosine kinase receptors, VEGFR-1 (Fms-like tyrosine kinase: Flt-1) and VEGFR-2 (kinase domain-containing receptor: KDR/flk-1). Flt-1 is expressed in two forms via alternative splicing at the pre-mRNA level; a full-length, membrane-bound receptor, capable of transducing signal, and a truncated, soluble receptor (sFlt-1), capable of sequestering ligands or dimerizing with full-length receptors and preventing signal transduction. It has been shown that VEGF is overexpressed in the hyperthecotic ovarian stroma of polycystic ovaries (PCO) [11, 12]. In addition, several investigators reported VEGF to be increased in serum and follicular fluid of PCOS women [5, 13, 14]. Moreover, sFlt-1, the soluble receptor for VEGF, has been shown to be decreased in serum of PCOS women undergoing controlled ovarian stimulation, contributing to increased VEGF bioavailability [15]. Several studies have demonstrated increased vascularity in the ovarian stroma of PCOS women as measured by Doppler blood flow velocities [13, 16, 17]. This increased ovarian vascularity was shown to correlate with increased serum VEGF levels in PCOS women [13, 18], further supporting the notion that VEGF contributes to the vascular changes observed in PCO.

PlGF displays 53% homology with VEGF and is a member of the VEGF family which also includes VEGF-A, VEGF-B, VEGF-C, VEGF-D and VEGF-E. It encodes four isoforms (PlGF 1–4), composed of 131, 152, 203 and 224 amino acids after the removal of signal peptide (18 amino acids residues in length), respectively [19]. While other members of the VEGF family can bind both Flt-1 and Flk-1 receptors, PlGF binds exclusively Flt-1 receptor [20] with high affinity compared to VEGF-A and VEGF-B, the other members of the family able to specifically bind Flt-1 [21]. In cells co expressing VEGF and PlGF mRNA, a heterodimeric VEGF/PlGF protein has been described, in addition to the VEGF and PlGF homodimeric forms. This VEGF/PlGF heterodimer has been shown to promote capillary growth *in vivo* [22].

The role of PlGF in PCOS pathophysiology has not been previously investigated. We hypothesized that women with PCOS may have elevated serum and/or follicular fluid PlGF levels, which would be consistent with the increased levels seen in its family member, VEGF. Accordingly, the primary aim of our study was to investigate PlGF and sFlt-1 dynamics in serum and follicular fluid of women with PCOS as compared to women without PCOS (serving as controls) during controlled ovarian stimulation (COS).

## Methods

### Study subjects

We prospectively enrolled 14 PCOS women from women undergoing controlled ovarian stimulation (COS) in preparation for IVF or ICSI as previously described [6]. Diagnosis of PCOS was made according to the Rotterdam consensus [23]. All 14 PCOS women had oligo- or amenorrhea and at least 12 follicles 2-9 mm in diameter per ovary. Ten of the women had hyperandrogenemia and/or hyperandrogenism, and four had normal serum androgen levels and no clinical hyperandrogenism. Secondary causes of androgen excess and anovulation were excluded. The indications for COS in the PCOS group were male factor (7 women), ovulatory dysfunction after failed superovulation induction and intrauterine inseminations (4 women) and tubal factor (3 women). Fourteen non-PCOS control women undergoing COS in preparation for IVF or ICSI were matched to PCOS women by age, BMI and use of GnRH agonist/antagonist prior to enrollment. Inclusion criteria for control non-PCOS women were normal ultrasonic ovarian morphology, normal ovulatory cycles and no endocrine abnormalities. Inclusion criteria for all study women were ages between 20–38, adequate visualization of ovaries on transvaginal ultrasound and no hormonal treatment i.e. OCPs. Exclusion criteria were diminished ovarian reserve or endometriosis. The indications for COS in the control group were male factor (9 women) or tubal factor infertility (5 women). Patient clinical characteristics are given in Table 1. This study was approved by Maimonides Medical Center institutional review board. Written informed consent was obtained from all participating women.

**Table 1:** **Patient clinical data**

	PCOS	Non-PCOS	P value
*Age* (*years*)	30.1 ± 4.4	30.8 ± 3.7	NS
*Body mass index* (*Kg*/*m* ^*2*^)	25.5 ± 5.6	24.9 ± 3.9	NS
*Serum AMH* (*ng*/*ml*)	8.0 ± 6.5	3.1 ± 1.5	0.003
*Day 3 FSH* (*mIU*/*ml*)	5.2 ± 1.9	6.0 ± 2.7	NS
*Total gonadotropin dose* (*IU*)	2057 ± 868	2767 ± 1786	NS
*Estrogen on day of hCG* (*pg*/*ml*)	2819 ± 1158	2898 ± 860	NS
*No. of aspirated oocytes*	17.4 ± 10.5	12.6 ± 7.2	NS
*Fertilization rate* (%)	67.1	64.8	NS
*Clinical pregnancy rate* (%)	50.0	42.9	NS

Note: PCOS, polycystic ovarian syndrome; OHSS, ovarian hyperstimulation syndrome; NS, not significant; values are given as mean “ standard deviation or percentages. P-value <0.05 was considered statistically significant.

Women were treated using a stimulation protocol which included either down regulation using a GnRH agonist in a long protocol (n = 8 for each group) or a GnRH antagonist to prevent premature ovulation (n = 6 for each group). Ovarian stimulation was performed using a combination of recombinant FSH and HMG. The standard stimulation protocol was modified when there was risk of ovarian hyperstimulation or previous history of poor response. Follicular monitoring by ultrasound and blood sampling for estradiol levels were performed every 1–3 days. After the first 3–5 treatment days, the daily dose could be adjusted based on the follicular development and estradiol levels. When at least six follicles with a diameter of 16 mm were detected, either 5,000 or 10,000 IU hCG was administered, depending on the estimated risk for hyperstimulation. Oocytes were retrieved under transvaginal sonographic guided needle puncture 35 hours following hCG administration. Clinical pregnancy was defined as the presence of a gestational sac on ultrasound performed at 6 weeks after embryo transfer. Fertilization rate was calculated as the number of fertilized oocytes divided by the number of oocytes retrieved. The clinical pregnancy rate (CPR) was calculated as the number of clinical pregnancies divided by the number of embryo transfer procedures.

### Collection of blood and follicular fluid

Blood samples were obtained by venipuncture on cycle day 3, day of hCG administration and day of oocyte retrieval. For AMH determination, blood samples were collected within the period of 3 months prior to controlled ovarian stimulation. After collection, the blood samples were allowed to clot at room temperature for 30 min, followed by centrifugation at 1200 rpm for 10 min. Serum was stored in aliquots at −80°C until assayed. For follicular fluid collection, follicles with a diameter of >16 mm were aspirated. Only the first clear follicular fluid aspirate associated with the presence of an oocyte, without blood or flushing solution, was used for analysis. After removal of the oocyte, the fluid was centrifuged at 1200 rpm for 10 min to remove granulosa cells and debris. The supernatant was divided into aliquots and stored at −80°C until assayed.

### PlGF, sFlt-1 and AMH ELISA assays

PlGF and sFlt-1 concentrations of serum and follicular fluid samples were determined by ELISA according to the manufacturer’s protocols (R&D, Minneapolis, MN), having a sensitivity of 7.0 pg/mL and 3.5 pg/mL, respectively. The intra- and inter-assay coefficients of variation for PlGF and sFlt-1 ELISAs were 7.0% and 11.2%, and 3.2% and 5.5%, respectively. AMH concentration of serum samples was determined according to the manufacturer’s protocols (Beckman and Coulter, Brea, CA). The assay sensitivity was 0.16 ng/ml. The intra- and inter-assay coefficients of variation were 4.3% and 7.4%, respectively. All assays were performed in duplicate.

### Statistics

A power analysis powered for a two-tailed t-test was carried out prior to subject enrollment to determine the minimum required sample sizes needed to detect a 1.5-fold (assumed) statistically significant difference in growth factor between cases (PCOS) and controls. The required level of significance was at least 95% (p = 0.05), and the required power of the test was at least 90% (p = 0.10). Based on this calculation the required sample sizes were n = 14 for each group. Data were analyzed by Student’s t-test or the Mann–Whitney test, as appropriate. Results are expressed as mean +/- standard error of the mean (SEM). Correlations between PlGF or sFlt-1 and various parameters were performed using Pearson correlation tests. SigmaStat (SPSS Science, Chicago, IL) was used for statistical analysis. All significance tests were two-tailed and P-value <0.05 was considered to be statistically significant.

## Results

Clinical characteristics and data of our study subjects are shown in Table 1. As expected, PCOS women had a significantly greater serum AMH level than the non-PCOS control group (8.0 ± 6.5 ng/ml vs. 3.1 ± 1.5 ng/ml, p = 0.003) (Table 1).

**Figure 1:**
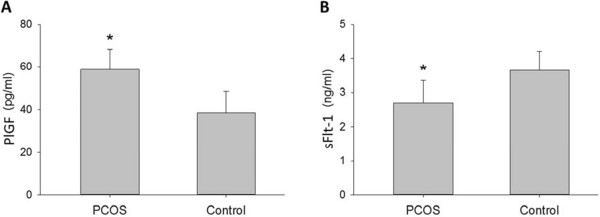
**Follicular fluid PlGF and sFlt**-**1 concentrations.** Follicular fluid concentration of PlGF (pg/ml) **(A)** and sFlt-1 (ng/ml) **(B)** in PCOS (polycystic ovarian syndrome) and non-PCOS women undergoing controlled ovarian stimulation. Data are presented as mean ± standard deviation. *p < 0.05 for PCOS vs. non-PCOS women.

Follicular fluid PlGF protein concentration on day of oocyte retrieval was increased approximately 1.5-fold in PCOS women compared with non-PCOS controls (58.7 ± 19.5 vs. 38.6 ± 14.0 pg/ml, respectively, p = 0.006) (Figure 1A). No differences were observed between PCOS and non-PCOS women in serum levels of PlGF at all three measured time points throughout controlled ovarian stimulation (Table 2).

**Table 2:** **Serum PlGF** (**pg**/**ml**) **in PCOS and non**-**PCOS women during controlled ovarian stimulation**

Group	Day 3	hCG	Oocyte retrieval
*PCOS*	9.6 ± 1.1	10.7 ± 1.4	10.1 ± 1.9
*Control*	9.7 ± 1.4	10.8 ± 2.0	10.1 ± 2.0

Note: PCOS, polycystic ovarian syndrome; values are mean “ standard deviation.

**Figure 2:**
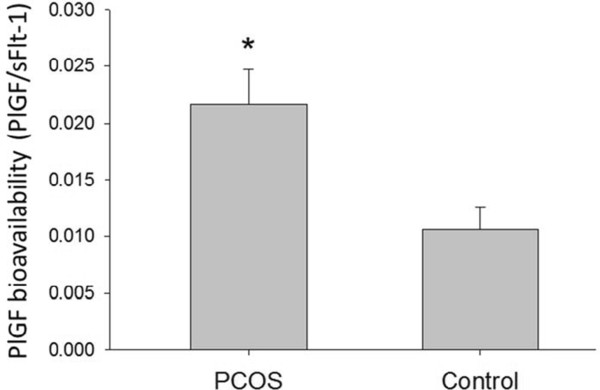
**Follicular fluid PlGF bioavailability is increased in PCOS.** PlGF bioavailability (PlGF/sFlt-1 ratio) in follicular fluid (ng/ml) of PCOS (polycystic ovarian syndrome) and non-PCOS women undergoing controlled ovarian stimulation. Data are presented as mean ± standard deviation. *p < 0.01 for PCOS vs. non-PCOS women.

**Figure 3:**
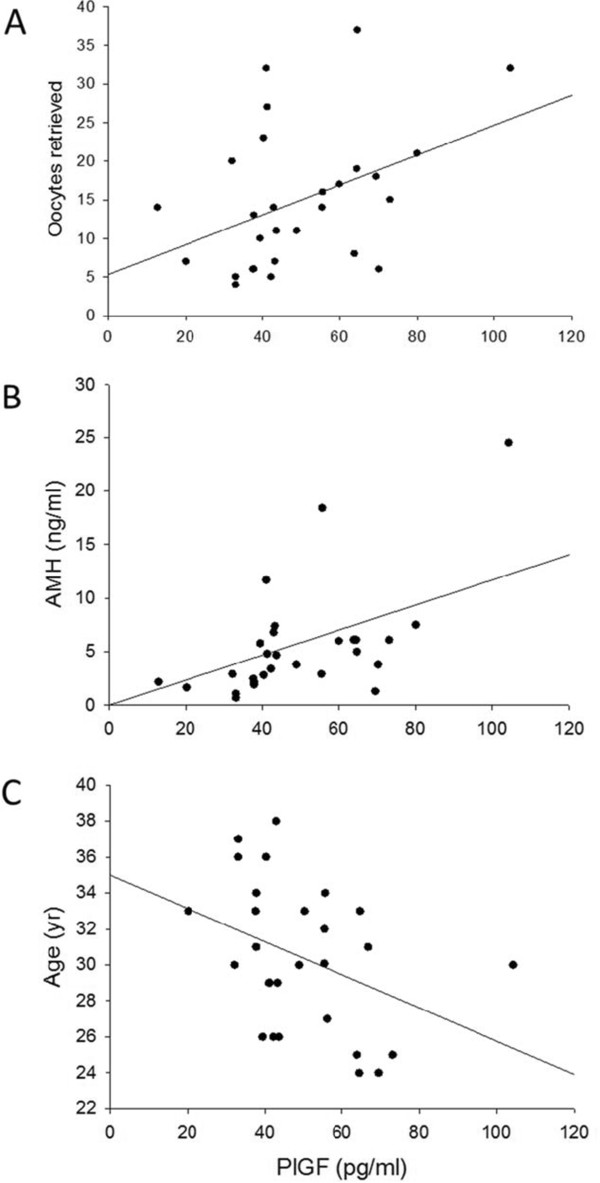
**Follicular fluid PlGF correlates with oocyte yield**, **AMH and age.** Correlations between follicular fluid levels of PlGF and **(A)** number of oocytes retrieved, **(B)** AMH or **(C)** age in women with polycystic ovarian syndrome or controls. Positive correlations were found between PlGF and number of oocytes retrieved (r = 0.41, p = 0.03) and between PlGF and AMH (r = 0.60, p = 0.001). PlGF and age correlated inversely (r = −0.38, p = 0.04).

Follicular fluid concentration of sFlt-1 was decreased 1.4-fold in PCOS compared with non-PCOS women (2.7 ± 0.6 vs. 3.7 ± 0.5 ng/ml, respectively, p = 0.04) (Figure 1B). Similarly, no differences were noted between PCOS and non-PCOS women in sFlt-1 serum levels at all three measured time points throughout controlled ovarian stimulation (Table 3). Since sFlt-1 binds PlGF, reducing its free levels and preventing its signal transduction, we calculated PlGF bioavailability as a ratio of PlGF to sFlt-1 (PlGF/sFlt-1). PlGF bioavailability in follicular fluid was significantly greater (2-fold) in PCOS women compared with non-PCOS controls (0.022 vs. 0.01, respectively, p < 0.01) (Figure 2).Pearson correlation analysis was performed to evaluate for correlations between PlGF or sFlt-1 concentration and various stimulation cycle parameters. Follicular fluid PlGF correlated positively with number of oocytes retrieved (r = 0.41, p = 0.03) and AMH (r = 0.60, p = 0.001), and inversely with age (r = −0.38, p = 0.04) (Figure 3). Follicular fluid PlGF was not found to correlate with total gonadotropin dose administered (r = −0.29, p = 0.15), peak estrogen level (r = 0.16, p = 0.43) or day 3 FSH (r = 0.09, p = 0.66). With regards to stimulation cycle outcomes, no correlation was found between follicular fluid PlGF and fertilization rate (r = −0.20, p = 0.34). Moreover, no difference was noted in follicular fluid PlGF level between pregnant and non-pregnant women (49.8 vs. 43.8 pg/ml, p = NS). No correlations were found between follicular fluid sFlt-1 and either age, AMH, day 3 FSH, total gonadotropin dose, peak estrogen level, number of oocytes retrieved, fertilization rate or clinical pregnancy rate (data not shown).

**Table 3:** **Serum sFlt**-**1** (**ng**/**ml**) **in PCOS and non**-**PCOS women during controlled ovarian stimulation**

Group	Day 3	hCG	Oocyte retrieval
*PCOS*	55.9 ± 9.3	56.3 ± 7.9	57.2 ± 10.7
*Control*	58.1 ± 8.0	57.6 ± 7.4	55.2 ± 8.6

Note: PCOS, polycystic ovarian syndrome; values are mean “ standard deviation.

## Discussion

This study is the first to report levels of PlGF in serum and follicular fluid of women with PCOS undergoing controlled ovarian stimulation. These data demonstrate that follicular fluid PlGF concentration is increased while concentration of its soluble receptor sFlt-1 is decreased, resulting in increased PlGF bioavailability in women with PCOS. In addition, this study provides the first evidence that follicular fluid PlGF is associated with serum AMH and number of aspirated oocytes.

VEGF has been shown to be increased in ovarian tissue [11, 12] as well as serum and follicular fluid [5, 13–15] of PCOS women compared with non-PCOS controls. In addition, sFlt-1, the soluble receptor for VEGF and PlGF, has been reported to be decreased in serum and follicular fluid of PCOS women undergoing controlled ovarian stimulation, contributing to increased VEGF bioavailability [15]. The increased serum VEGF levels in PCOS were shown to correlate with increased ovarian vascularity in these women [13, 18], while laparoscopic ovarian drilling has been shown to reduce Doppler indices of ovarian stromal blood flow [24] with a concomitant reduction in circulating VEGF levels [25], supporting the notion that VEGF contributes to the vascular changes observed in PCO. Our results demonstrate that follicular fluid PlGF, another important VEGF family member, is increased in PCOS and suggest a role for PlGF overactivity in the pathogenesis of PCOS. Additional research is warranted to investigate the relative contribution of PlGF to the increased ovarian vascularity and pathophysiology of PCOS. In contrast to VEGF, which is increased in both serum and follicular fluid of PCOS women [5, 13–15], PlGF was not increased in serum of PCOS women in our study. This difference may be due to increased VEGF production in PCOS by non-ovarian sources. In support of this, obesity and insulin resistance, which are closely associated with PCOS, are characterized by VEGF overproduction by adipose, vascular and bone-marrow derived cells [26, 27]. An alternative explanation for the increased serum VEGF, but not serum PlGF, in PCOS women may be increased ovarian production of VEGF relative to PlGF. However, the follicular fluid:serum ratio of PlGF was found to be 4 to 6 fold in our study, which is comparable to previously reported follicular fluid:serum ratio of VEGF (3 to 4 fold) [5], making this explanation less likely.

AMH, or mullerian-inhibiting substance (MIS), is widely considered a highly sensitive marker of ovarian reserve, and has been shown to correlate with ovarian response to stimulation during ART, as well as the risk of OHSS [28, 29]. In addition, AMH correlates inversely with age, and its levels become undetectable in the serum of menopausal women [30]. Our results revealed that follicular fluid PlGF correlated positively with serum AMH and number of oocytes retrieved while it correlated inversely with age, suggesting that follicular fluid PlGF, similarly to AMH, may be associated with ovarian response to stimulation. While no studies, to the best of our knowledge, have previously reported on associations between follicular fluid PlGF and ART cycle stimulation/outcomes, previous observations showed that follicular fluid VEGF correlates positively with age and negatively with number of oocytes retrieved and pregnancy rates [31, 32]. These data suggest that PlGF and VEGF, while belonging to the same VEGF family, may have different biological roles in the local ovarian microenvironment. Further studies are needed to confirm our data and investigate potential associations between PlGF and ART outcomes.

Several lines of evidence indicate that PlGF is a potent pro-angiogenic factor which contributes to pathological angiogenesis. PlGF and VEGF have been demonstrated to have similar potency in stimulating tissue factor production and chemotaxis in monocytes [33]. In addition, PlGF has been shown to stimulate angiogenesis and collateral growth in ischemic heart and limb with efficiency at least comparable to VEGF [34]. Moreover, Suzumori et al. have reported that PlGF concentration is increased in peritoneal fluid of women with endometriosis, and suggested that it may contribute to the pathological neovascularization characteristic of endometriotic lesions [35]. Since VEGF is considered a key mediator of ovarian hyperstimulation syndrome (OHSS) by increasing vascular permeability, its overexpression in PCOS has been suggested to contribute to the increased risk of OHSS seen in these patients [36]. As the risk of OHSS is known to be greater in women receiving GnRH agonist protocol as compared to GnRH antagonist protocol, we matched PCOS women to non-PCOS women according to study protocol, eliminating this potential bias. In our study, follicular fluid PlGF was increased in PCOS and positively correlated with the number of oocytes retrieved i.e. ovarian stimulation. Thus, it is interesting to speculate whether PlGF may play a similar role to VEGF in OHSS. Indeed, transgenic mice overexpressing PlGF in skin under the control of keratin-14 promoter showed a substantial increase in number, branching and size of dermal blood vessels, together with enhanced vascular leakiness [37], while inhibition of PlGF was associated with reduced plasma extravasation in pathological conditions [38]. Moreover, the gonadotropin LH, which is well-known to stimulate VEGF leading to OHSS [39], has been shown to increase the follicular fluid PlGF/sFlt-1 ratio in women undergoing ovarian stimulation [40]. Further studies are needed to evaluate the potential role of PlGF in OHSS pathogenesis and examine its utility as a predictor for early and/or late OHSS.

In the current study, PlGF concentration was 4 to 6-fold greater in follicular fluid compared with serum, suggesting that the ovary is the primary site of production of follicular fluid PlGF. Consistent with our data, Gutman et al. reported higher levels of PlGF in follicular fluid compared with plasma (where levels were undetectable) in women undergoing IVF [40]. A possible explanation for the differences in ability to measure PlGF in the circulation between the studies is the different type of sample medium used (i.e. serum vs. plasma). Follicular fluid is the product of diffusion of blood constituents across the blood-follicle barrier and of molecules secreted by granulosa and theca cells. Although it is unknown which ovarian compartment may be responsible for PlGF production, there is a strong body of evidence that ovarian granulosa and endothelial cells are the main source of production of VEGF during hyperstimulation [36, 41], and it s reasonable to hypothesize that PlGF is produced by similar mechanisms. In-vitro and animal experiments are needed to clarify this point further.

## Conclusions

This is the first study to characterize serum and/or follicular fluid levels of PlGF in PCOS during controlled ovarian stimulation. Our study provides evidence that follicular fluid PlGF correlates with ovarian stimulation and that its bioavailability, as measured by the PlGF/sFlt-1 ratio, is increased in women with PCOS. Results of this study suggest that PlGF may play a role in the angiogenic dysregulation characteristic of PCOS and its predisposition to OHSS. Further studies are warranted to investigate these possibilities.
